# Resetting the Drift of Oxygen Vacancies in Ultrathin HZO Ferroelectric Memories by Electrical Pulse Engineering

**DOI:** 10.1002/smsc.202400223

**Published:** 2024-07-30

**Authors:** Atif Jan, Stephanie A. Fraser, Taehwan Moon, Yun Seong Lee, Hagyoul Bae, Hyun Jae Lee, Duk‐Hyun Choe, Maximilian T. Becker, Judith L. MacManus‐Driscoll, Jinseong Heo, Giuliana Di Martino

**Affiliations:** ^1^ Department of Materials Science and Metallurgy University of Cambridge Cambridge CB3 0FS UK; ^2^ Department of Electrical and Computer Engineering University of Southern California Los Angeles CA 90089 USA; ^3^ Samsung Advanced Institute of Technology Samsung Electronics Suwon‐si 16678 Korea; ^4^ Department of Electronics Engineering Jeonbuk National University Jeonju‐si 54896 Korea

**Keywords:** dark‐field spectroscopies, domain wall motions, fatigues, HZO ultrathin ferroelectric random‐access memories, oxygen vacancy migrations, Raman and photoluminescence, wake‐up

## Abstract

Ferroelectric HfO_2_‐based films incorporated in nonvolatile memory devices offer a low‐energy, high‐speed alternative to conventional memory systems. Oxygen vacancies have been rigorously cited in literature to be pivotal in stabilizing the polar noncentrosymmetric phase responsible for ferroelectricity in HfO_2_‐based films. Thus, the ability to regulate and control oxygen vacancy migration in operando in such materials would potentially offer step changing new functionalities, tunable electrical properties, and enhanced device lifespan. Herein, a novel in‐ operando approach to control both wake‐up and fatigue device dynamics is reported. Via clever design of short ad hoc square electrical pulses, both wake‐up can be sped up and both fatigue and leakage inside the film can be reduced, key factors for enhancing the performance of memory devices. Using plasmon‐enhanced photoluminescence and dark‐field spectroscopy (sensitive to <1% vacancy variation), evidence that the electrical pulses give rise to oxygen vacancy redistribution is provided and it is shown that pulse engineering effectively delays wake‐up and reduces fatigue characteristics of the HfO_2_‐based films. Comprehensive analysis also includes impedance spectroscopy measurements, which exclude any influence of polarization reversal or domain wall movement in interpretation of results.

## Graphical abstract

Short electrical pulses to “reset” *V*_O_ migration in ultrathin ferroelectric oxides are engineered. Electrical pulse modification regulates wake‐up, reduces fatigue, and leakage. Reset waveforms exhibit ability to control wake‐up by ≈4× to ≈24×, retard fatigue by 2× to 4×, and minimize leakage by ≈60%. Photoluminescence and dark‐field spectroscopy show strong evidence that this is linked to film *V*_O_ control.

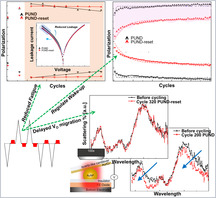

## 1 Introduction

Ferroelectric (FE) thin films have been extensively studied in nonvolatile semiconductor devices, with a focus on its possible uses in FE random‐access memory (FeRAM)^[^ ^1^, ^2^, ^3^ ^]^ and FE field‐effect transistors (FeFETs).^[^ ^4^ ^]^ The unexpected presence of ferroelectricity in thin films based on HfO_2_ was first observed in 2007 by Böscke et al.^[^ ^5^ ^]^ Since then, further investigations have been conducted on HfO_2_ and several binary oxide^[^ ^6^ ^]^‐based FeRAMs and FeFETs have been realized, with the objective of surpassing conventional memory technologies in terms of low voltage/power requirements, fast writing speed, and endurance.^[^ ^7^, ^8^ ^]^ Numerous experimental and theoretical investigations on binary oxide FE materials have been published since, comprehensively discussing the impact of doping, mechanical stress, interfaces, FE film thickness, and oxygen vacancies on device performance.^[^ ^5^, ^9^, ^10^, ^11^, ^12^, ^13^ ^]^

Recent advances have made doped binary oxides with the composition Hf_0.5_Zr_0.5_O_2_ (HZO) most desirable for ultrathin‐film microelectronics applications.^[^ ^14^, ^15^ ^]^ The metastable polar noncentrosymmetric orthorhombic (o) phase, responsible for ferroelectricity, is often reportedly stabilized by oxygen vacancies among other factors^[^ ^16^, ^17^, ^18^, ^19^, ^20^, ^21^ ^]^ and those are widely acknowledged as crucial for the functionality of devices. Jan et al.^[^ ^11^ ^]^ comprehensively elucidated the role of oxygen vacancies in the precise atomistic principles that underlie the phenomena of wake‐up and fatigue, known to cause device performance variation over its lifetime which is detrimental for the performance of HfO_2_‐based nonvolatile memories and low‐power logic devices. In fact, during field cycling of such devices, migration of oxygen vacancies (*V*_O_) in FE binary oxides preferentially induces phase change inside the film from monoclinic (m) and tetragonal (t) phase into o‐phase, resulting in higher polarization (*P*).^[^ ^11^, ^22^ ^]^ An abundance of oxygen vacancies results in accelerated fatigue and also influences the wake‐up effect.^[^ ^11^ ^]^ Recent reviews and perspectives label wake‐up and fatigue along with high coercive voltage as the principal obstacles in reliable operation of HfO_2_‐based FE devices^[^ ^23^, ^24^, ^25^, ^26^ ^]^ and therefore has attracted lot of research.

Li et al.^[^ ^27^ ^]^ in an earlier work showed an increase in the number fatigued domains formed La:HfO_2_ films, by modulating the switching voltage and pulse width. Nair et al.^[^ ^28^ ^]^ in their work, cleverly show that changing the switching waveform from triangular to a square pulse reduces the number of wake‐up cycles and increases the concomitant remnant polarization. Lenox et al.^[^ ^29^ ^]^ suggested an increase in device endurance by reducing duty cycle of switching waveform. However, none of the earlier works provides a holistic approach that can address wake‐up, fatigue, and leakage simultaneously and employ asymmetric switching voltages that can induce fatigue in the device by not switching polarization states completely.^[^ ^27^ ^]^ Furthermore, there is also a lack of in operando atomistic explanation of the impact of different voltage waveforms.

## 2 Results

The premise of this work is to engineer electrical pulses to “reset” (reverse the drift or redistribute) vacancies in order to modulate the wake‐up, reduce fatigue, and leakage in the FE film. We examine the effects of using low‐voltage, time‐limited, electrical (reset) pulses to in operando reset/redistribute oxygen vacancies and prove its crucial impact on manipulating wake‐up and retarding fatigue, hence influencing the lifespan and performance of the device. Unlike previous works,^[^ ^27^, ^28^, ^29^ ^]^ we do not change the frequency, duty cycle, or the voltage for switching between the two polarization states to address the aforementioned nonidealities. We employ PUND that remains constant throughout to switch between polarization states. The conventional PUND (or double‐wave method^[^ ^30^, ^31^, ^32^ ^]^) uses five consecutive triangular electrical pulses. The first prepolarization pulse (electric field (*E*)) sets all domains in “down” direction. In the subsequent sequence of four pulses, the subtraction of two consecutive positively (negatively) oriented pulses yields a semi *P*–*E* loop for *E* > 0 (*E* < 0) to extract the remanent polarization. We prove that employing additional low‐voltage, time‐limited, squared reset pulses between the triangular pulses resets/redistributes oxygen vacancies, subsequently impacting polarization, wake‐up, fatigue, and device lifetime.

**Figure 1:**
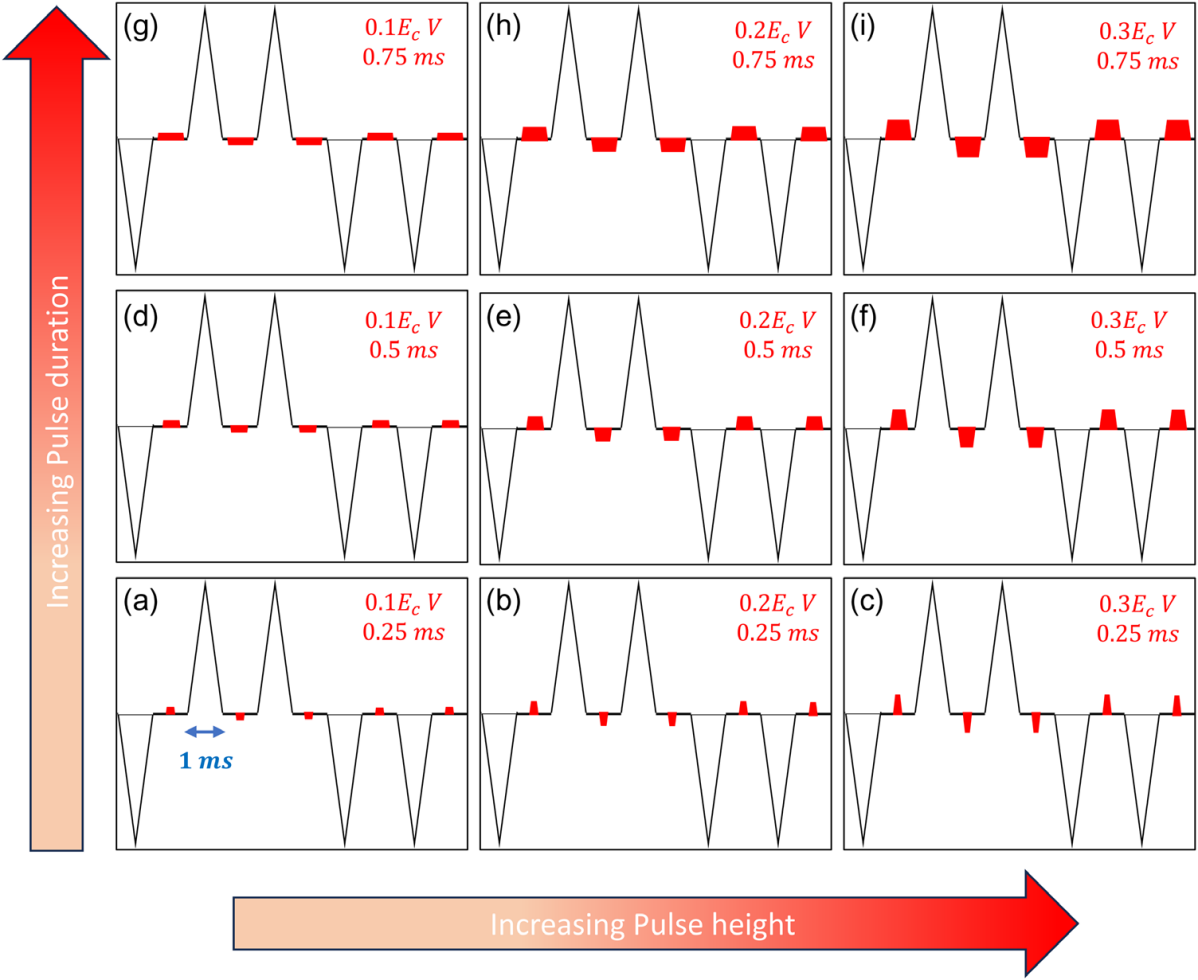
Pulse‐engineered PUND‐reset waveforms. a–i) PUND‐reset pulses. Every square reset pulse is fixed in the opposite direction of the preceding triangular PUND pulse. The amplitude of reset pulse is in the range 0.1*E*_C_–0.3*E*_C_. The duration of the pulse varies from 0.25 to 0.75 ms. All waveforms are at 1 kHz (1 ms duration).

The reversible nature of oxygen vacancy movement has been previously studied in an epitaxial HZO model system grown by pulsed laser deposition on SrTiO_3_/La_1−*x*_Sr_*x*_MnO_3_ (LSMO) single‐crystal substrates and covered with LSMO top electrodes (TEs).^[^ ^33^ ^]^ In this epitaxial HZO model system, it has been found that phase transformation is intertwined with FE switching and it was shown that the HZO layer acts as a fast conduit for oxygen vacancies between LSMO electrodes even at millisecond timescales.^[^ ^33^ ^]^ In this study, we focus on 5 nm‐thick polycrystalline FE HZO films grown by atomic layer deposition (ALD), with coercive and breakdown fields *E*_C_ ≈ 1.9 MV cm^−1^ and *E*_BD_ ≈ 5–8 MV cm^−1^, respectively. Thus, we cycle the device from ±2 V (±4 MV cm^−1^), to limit the highest field to 4 MV cm^−1^, which is sufficiently below *E*_BD_ and above *E*_C_ (Supporting Information (a)). To reset *V*_O_ migration (from neighbouring sites), we modify the conventional PUND waveform by adding low‐voltage (0.1–0.3 V) and variable duration (0.25–0.75 ms) square pulses between the individual triangular PUND pulses (**Figure** **1**), in opposite directions to the preceding triangular pulse.

### 2.1 Electrical Characterization

The field cycling of a pristine FE film leads to an increase in the remnant polarization for the first ≈100–1000 cycles,^[^ ^11^, ^22^, ^34^, ^35^ ^]^ a phenomenon called wake‐up. It entails the redistribution of vacancies inside the FE film, leading to phase change from predominant m/t‐phase to FE o‐phase HZO.^[^ ^11^, ^17^ ^]^

**Figure 2:**
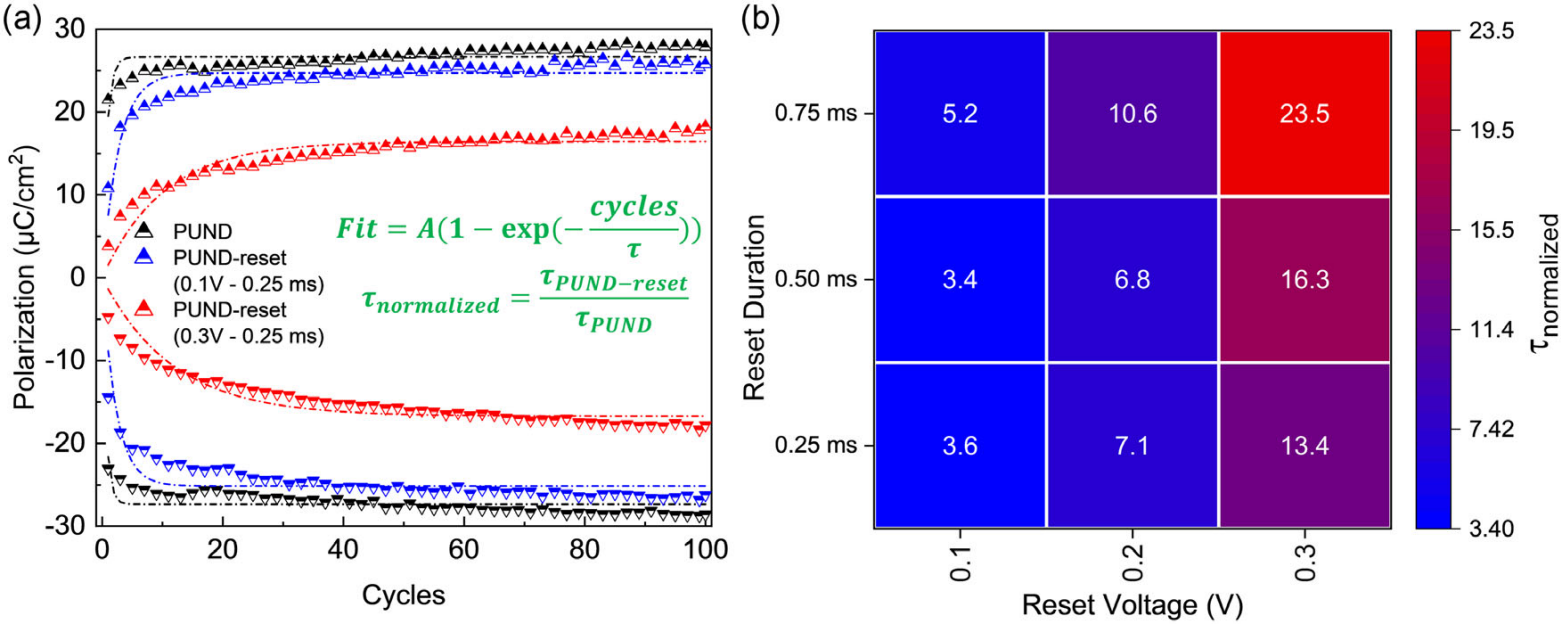
Wake‐up effects with PUND‐reset waveform. a) Polarization versus switching cycles for PUND (black) and two representative PUND‐reset (blue: 0.1 V/0.25 ms, red: 0.3 V/0.75 ms) pulses. Symbols are for data points and dashed lines for the fit. All 9 PUND‐reset profiles can be found in Supporting Information (b). The characteristic parameter for PUND‐reset normalized to that of the PUND is defined as *τ*_normalized_ for comparing delay in time to wake‐up. b) Heatmap showing the *τ*_normalized_ time constants for all 9 PUND‐reset.

We cycle pristine devices with ten different switching pulse profiles, that is, 1 standard PUND pulse and 9 PUND‐reset pulses (Figure 1), for a total of 100 cycles. We then fit the progression of polarization versus cycles with the empirical relation, $A(1-\exp\left(-\frac{\text{cycles}}{\tau }\right))$, where *τ* represents the characteristic parameters for the standard PUND ($\tau_{\text{PUND}}$) and the investigated 9 PUND‐reset pulses ($\tau_{\text{PUND}-\text{reset}}$), respectively (**Figure** **2**a). The polarization versus cycles could best be fit with an exponential curve, where *τ* is an estimate of the number of cycles till the polarization wake‐up is achieved. The parameter A is the “final polarization value” of the fit for the first 100 cycles. We define a figure of merit, $\tau_{\text{normalized}}=\frac{\tau_{\text{PUND}-\text{reset}}}{\tau_{\text{PUND}}}$, to show the variance in delay of wake‐up for all devices and display it as a heatmap (Figure 2b). For all nine characteristic parameters, we highlight that an increase in the reset pulse amplitude (0.1–0.3 V) and time (0.25–0.75 ms) significantly increases the time to wake‐up (≈4× to ≈24×), hence considerably delaying wake‐up processes. Given that wake‐up has been ascribed to *V*_O_ migration inside the film leading to phase change of the oxide material,^[^ ^11^, ^33^, ^36^ ^]^ the direct correlation of the in situ electrical reset of *V*_O_ migration with the observed delay in wake‐up would not be unexpected. Interestingly, and technologically rather relevant, we can speed up wake‐up with reset pulses applied in the same direction as that of the preceding triangular pulse in the PUND‐waveform. Up to a 1.25× decrease in cycles to wake‐up is observed (Figure S3, Supporting Information (b)) in devices cycled with PUND‐reset waveforms, compared to PUND only.

**Figure 3:**
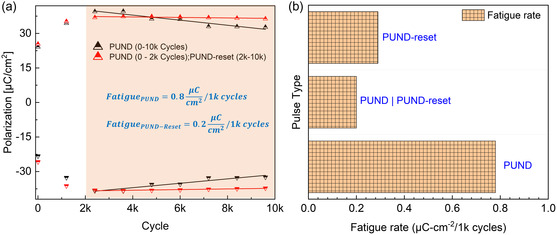
Reduced fatigue with PUND‐reset waveforms. a) Device with common wake‐up, that is, both cycled with PUND for 2000 cycles until wake‐up. Post 2000 cycles device cycled with PUND (black) and 0.1 V–0.25 ms PUND‐reset (red). The solid lines show linear fit (2000–10 000 cycles, after wake‐up (shaded area)) to extract slope of fatigue for each device. b) Fatigue rate (slope of polarization versus cycles during fatigue) for devices cycled with: PUND only (0.8 μC cm^−2^ 1k cycles^−1^), PUND (common wake‐up), PUND‐reset for the rest of cycles (0.2 μC cm^−2^ 1k cycles^−1^), and PUND‐reset only (0.3 μC cm^−2^ 1k cycles^−1^).

The further cycling eventually sets the films into a fatigue, where the remnant polarization reduces. The microscale principles setting FE films into fatigue are extensively debated^[^ ^37^, ^38^ ^]^ and are largely described with three mechanisms: 1) volume effect; 2) domain effect; and 3) grain boundary effect.^[^ ^37^ ^]^ Common to all of them is that mobile charged defects (*V*_O_) stabilize a given domain configuration, thereby generating an internal bias field that counteracts the polarization reversal when an electric field is applied. The defect generation (*V*_O_) during memory state alternation and charge injection across electrodes affect the switching properties of the FE film, causing domain pinning and a drop in *P*_r_.^[^ ^39^ ^]^ Therefore, cycling the devices with PUND‐reset pulses potentially creates a reversal to the formation kinetics of *V*_O_ and may lead to a meaningful impact on retarding fatigue. To test the effect of reset pulses on the *V*_O_ reversal during fatigue, we compare devices (over 10 000 cycles) biased with 1) standard PUND throughout (**Figure** **3**a, black); and 2) with standard PUND during wake up (<2000 cycles) and PUND reset (0.1 V, 0.25 ms) during fatigue (>2000 cycles) (Figure 3a, red). This allows for both devices to go through the same wake‐up process, such that fatigue is observed independent of wake‐up. The device cycled with standard PUND pulses evidently undergoes a more aggressive fatigue when compared against PUND‐reset (Figure 3a). To quantify the reduced P variability during fatigue, we used a linear fit of polarization versus cycles. The slope of this fit is 0.8 μC cm^−2^ 1000 cycles^−1^ for standard PUND‐cycled devices and 0.2 μC cm^−2^ 1000 cycles^−1^ for PUND‐reset cycled device, resulting in a *P* variability already reduced by 400% when the smallest and shortest (0.1 V, 0.25 ms) reset pulses are used rather than the standard PUND pulses, thereby showing potential to extend the lifetime by a factor of 4 (Figure 3b). Devices cycled with PUND and PUND‐reset throughout, without a common wake‐up, show a similar improvement in fatigue characteristics (Supporting Information (c)). Devices with common wake‐up cycled 50k times also show similar advantages (extending lifetime by a factor of 2) in fatigue reduction (Supporting Information (c)).

**Figure 4:**
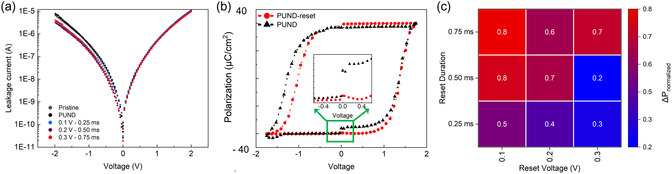
Leakage and polarization loss with PUND‐reset waveforms. a) Leakage current density curves for devices compared between pristine state and after cycling with PUND and PUND‐reset waveforms. b) *P*–*V* characteristics for PUND and PUND‐reset (0.3 V 0.5 ms) pulses. Inset: polarization loss as gap at 0 V. c) Heatmap shows a figure of merit Δ*P*_normalized_ defined as Δ*P* of PUND reset, normalized to conventional PUND. A reduction in leakage (Δ*P*_normalized_ < 1) is present for all PUND‐reset pulsed devices, with 0.3 V–0.5 ms showing least polarization loss.

In addition to proving the ability to manipulate wake‐up and reduce fatigue, we also studied the effect on device/film leakage when biased with PUND‐reset pulses. **Figure** **4**a exhibits the leakage current curves measured at ambient temperature. The leakage current of devices cycled with the PUND waveform (after 500 cycles) remains unchanged in comparison to the leakage in pristine devices. In contrast, leakage current evidently reduces for negative bias in devices cycled with PUND‐reset waveforms (after 500 cycles) as compared to pristine devices (59% difference between PUND and PUND‐reset leakage current at −2 V).

In usual polarization‐voltage (*P*–*V*) hysteresis loop measurements, it is typical to observe a gap at zero voltage (Supporting Information (d)). This gap at zero applied voltage is associated with the polarization loss caused by, for example, dielectric discharging, dielectric relaxation, fast decay, initial polarisation loss, and rapid depolarization.^[^ ^40^ ^]^ Furthermore, a decrease in the resistance of the capacitor (or increased leakage) is reported to increase the width of the gap, resulting in charge loss.^[^ ^41^ ^]^ A comparison of PUND with PUND‐reset pulses shows, rather interestingly, a decrease in the gap at 0 V (Figure 4b) in the latter case. From the gap at 0 V (Δ*P*) for PUND and PUND‐reset biased devices, we define a figure of merit Δ*P*_normalized_ =Δ*P*_PUND‐reset_/Δ*P*_PUND_ and display it as a heatmap for all PUND‐reset pulses (Figure 4c). Although a clear trend is not present, a reduction of the gap (Δ*P*_normalized_ < 1) is present for all PUND‐reset pulsed devices, with the 0.5 ms 0.3 V reset pulse showing the optimal reduction, with a remarkable ≈20% of the gap (Δ*P*_normalized_ = 0.21) otherwise obtained by the standard PUND pulse.

### 2.2 In situ Photoluminescence and Dark‐Field Spectroscopy

The direct correlation of the observed changes in device performances upon engineering of electrical pulses and the widely accepted effect of *V*_O_ migration inside the film on wake‐up/fatigue^[^ ^11^, ^33^, ^36^ ^]^ would hint at a *V*_O_ migration reversal induced by the reset pulses.

We selected the field intensity of the reset pulse <*E*_c_ to avoid any reversal of the polarization orientation of the FE oxide thin film. Given the preceding discussion, an assessment of the energy needed to move *V*_O_ is also needed while assessing the limits of the reset pulses between the individual triangular PUND pulses. The energy barrier for vacancy hopping between all‐nearest‐neighbor sites can be calculated for monoclinic (m) HfO_2_ using density functional calculations (DFT).^[^ ^42^ ^]^ In m‐HfO_2_, O atoms occur in threefold or fourfold coordinated sites, resulting in two nonequivalent oxygen vacancy sites. The activation barriers for migration between neighboring sites in m‐HfO_2_ are as low as 0.05–0.23 eV and higher for doubly positively charged vacancies.^[^ ^42^ ^]^ The migratory barrier in (Hf, Zr)O_2_ supercells is much smaller than the polarization transition barrier.^[^ ^43^, ^44^, ^45^, ^46^ ^]^ Therefore, an engineered electrical pulse amplitude ≥ activation barrier for migration between neighbouring sites. Although the DFT results in another study^[^ ^42^ ^]^ are instrumental in determining the lower limit of reset pulses, the upper limit remains contentious. In this study, the 0.1–0.3 V pulses are above the expected migration activation barrier; hence, *V*_O_ migration reversal is a possible mechanism. To prove this, we optically track *V*_O_ migration using plasmon‐enhanced spectroscopy (NPoM, Supporting Information (e)), a nondestructive technique able to track phase changes,^[^ ^11^ ^]^ ions intercalation, and variance in vacancies content with remarkable (<1%) sensitivity^[^ ^11^ ^]^ in thin (≈2–5 nm) films, with single‐switching cycle resolution.^[^ ^47^, ^48^, ^49^ ^]^ We incorporate optical measurements with slower measurements (≈1 Hz) that lead to overestimation of polarization.^[^ ^30^ ^]^ The NPoM device structure is also highly asymmetric, making it difficult to identify a “true” device area. Therefore, we chose to depict internal HZO variations as a function of charge variance instead of a finite polarization value. The charge integrated over current measurement period (*I*_P_–*I*_U_ is denoted as Δ*Q* (remanent polarisation) in general and Δ*Q*_0_ for first cycle. The change in polarization after sequential measurements is thus denoted by the ratio Δ*Q*/Δ*Q*_0_.^[^ ^11^ ^]^

**Figure 5:**
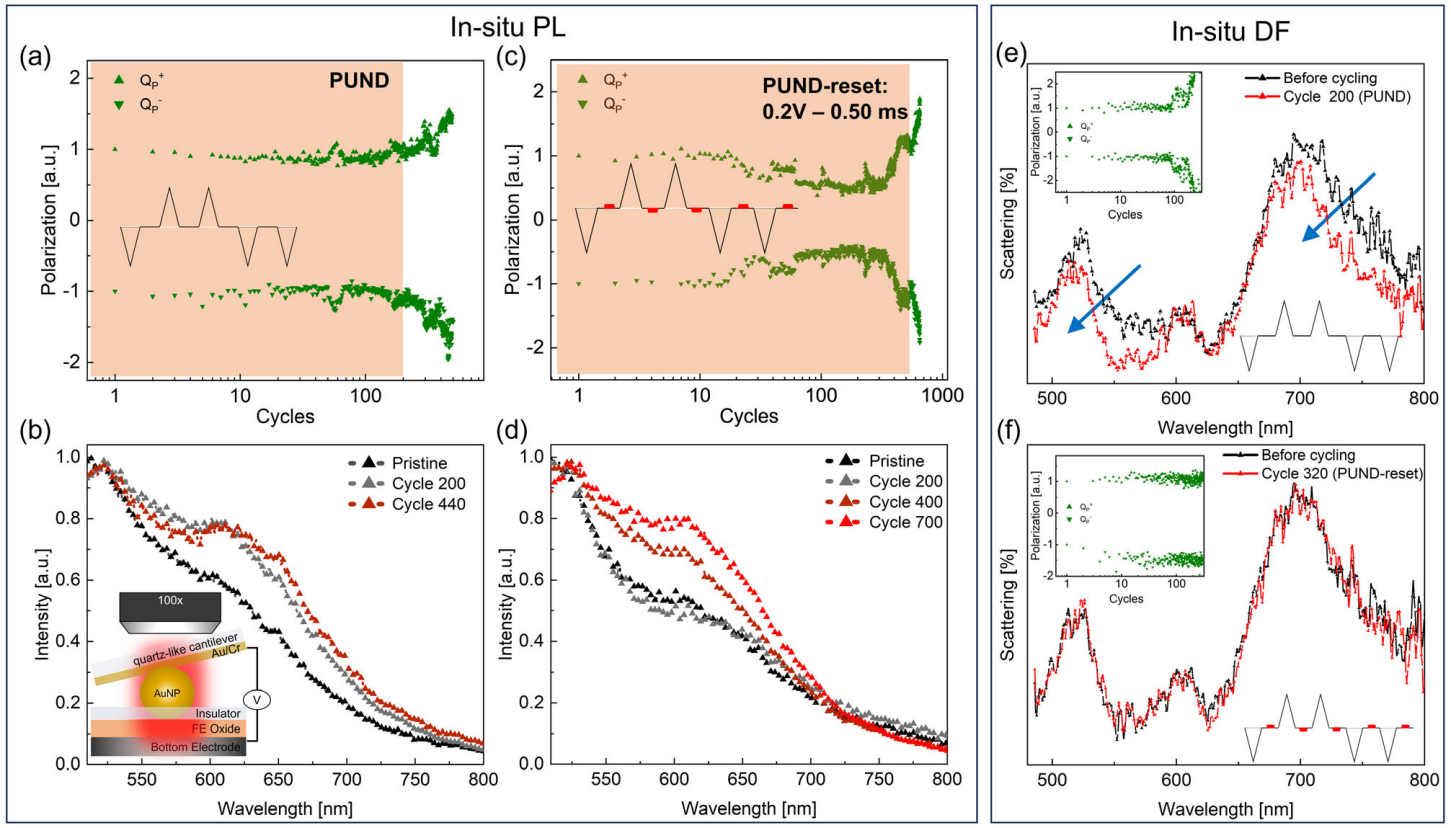
In situ spectroscopy with PUND‐reset waveforms. a,c) Electrical and b,d) PL traces of devices cycled in NPoM geometry. The insets in a, c show the PUND waveform used to cycle the devices; cycles to wake‐up are shaded in orange. The PL compares conventional PUND (a,b) and PUND reset (c,d) with a delayed response in wake‐up visible both in electrical (c) and optical trace (d). e,f) DF traces of devices cycled in NPoM geometry. The top insets show the electrical trace and bottom insets show the PUND waveform used to cycle. The DF peaks near 700 nm for conventional PUND (e) show left shift and lower intensity after 200 cycles indicative of vacancy defect generation compared to no change in (f).

We study the delay in wake‐up with PUND‐reset pulses by tracking changes both in defects levels—through photoluminescence (PL)—and dielectric permittivity—through dark‐field (DF)—upon wake‐up (**Figure** **5**). When cycling FE HZO films, we observe a considerable increase in the intensity of a small luminescence peak at 660 nm (already present in pristine devices) and a new emission at 565 nm upon wake‐up^[^ ^11^ ^]^ (Figure 5b,d). The two observed luminescence peaks at 565 and 660 nm have been previously shown to be strongly correlated with oxygen reduction in the FE oxide film.^[^ ^11^, ^50^ ^]^ Devices cycled with both standard PUND and PUND reset show an increase of *V*_O_ content, although the timing for this to occur differs considerably depending on the waveform used to cycle the device. FE HZO film cycled with standard PUND pulses, with a gold nanoparticle (AuNP) as TE, undergoes wake‐up from cycle 200 onward (Figure 5a). Optically, this is nicely linked to a change in PL (565 nm peak arising and 660 nm peak gaining strength) for cycles >200, confirming an increased density of *V*_O_ in the film at this stage of cycling (Figure 5b). On the contrary, a film cycled with a PUND reset (0.2 V–0.50 ms) has a much‐delayed wake‐up, only visible from cycle 400 onwards (Figure 5c). This is again manifested with the characteristic PL showing no visible change after 200 cycles (Figure 5d, grey), with a change being visible only after wake‐up is achieved, that is, for >400 cycles (Figure 5d, brown) and 3.5 times more cycles are needed to achieve the same PL intensity change observed for PUND‐cycled devices (Figure 5d, red), corroborating a delayed increase of *V*_O_ upon the presence of reset pulses.

DF spectra in NPoM configuration,^[^ ^47^, ^51^, ^52^ ^]^ that is, light scattered by NPoM (Figure 5b, inset) with optical modes highly dependent on dielectric constant changes in the switching film, show plasmonic resonant regions near wavelengths, *λ*_1_ = 520 nm, *λ*_2_ = 600 nm, and *λ*_3_ = 720 nm (Figure 5e–f). After cycling the device with PUND waveform for 200 cycles, clear decreases in signal intensity and slight blueshifts of the peak positions can be seen for *λ*_1_ and *λ*_3_ resonances (Figure 5e). The shift and intensity decrease is due to incorporation of vacancies into the film, as previously reported.^[^ ^11^ ^]^ No change is observed for device cycled with PUND‐reset waveform when cycled for more 320 cycles (Figure 5f). The DF analysis therefore corroborates the dynamics highlighted by PL and confirms our ability to delay morphological changes, that is, *V*_O_ drift, in the FE film.

### 2.3 Impedance Spectroscopy: Rayleigh Analysis

We can also exclude movement of domain walls and/or mobile interphase boundaries^[^ ^53^ ^]^ as the origin of the different wake‐up and fatigue behavior of the devices. To ascertain the impact of low‐voltage reset pulses on the movement of domain walls (see Supporting Information (f)), we perform impedance spectroscopy coupled with Rayleigh analysis, that quantifies the onset of domain wall motion.^[^ ^54^ ^]^ Impedance measurements were performed on FE HZO films prepoled at −2 V, over a frequency range of 10 Hz–1 MHz, with electric field amplitude (*E*_O_) varied from 10 to 500 mV root mean square (Supporting Information (g). The effective parallel capacitance ($C_{\mathrm{eff}}^{P}$) and the effective parallel resistance ($R_{\mathrm{eff}}^{P}$) of the HZO capacitor stack were estimated using standard equations listed in Supporting Information (h).

**Figure 6:**
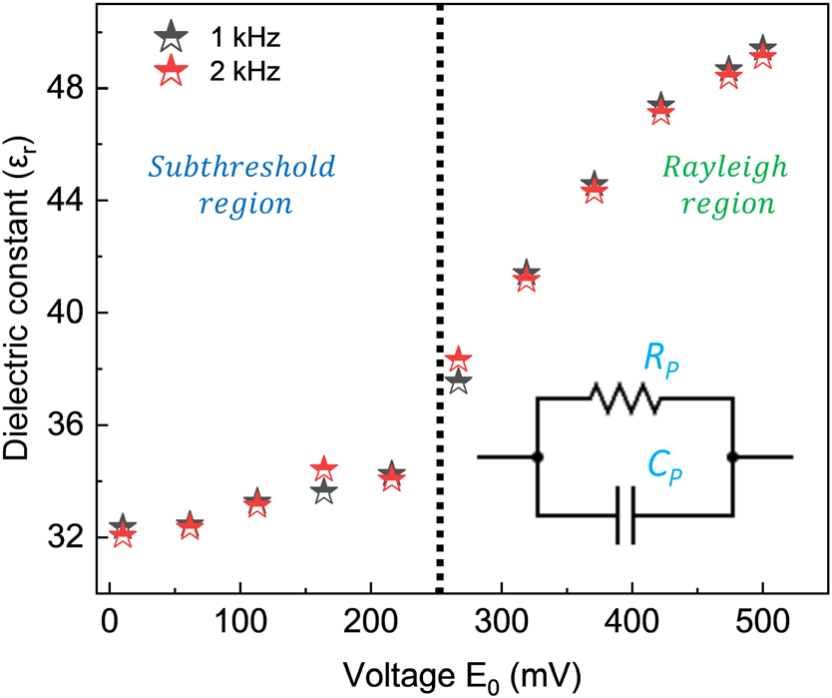
Impedance spectroscopy of 5 nm HZO FE capacitor stack. The effective dielectric permittivity of the HZO capacitor stack extracted at 1 and 2 kHz versus amplitude *E*_0_ of the AC electric field shows subthreshold (left of the black line) and Rayleigh region (right of the black line). The effective equivalent circuit to evaluate the measured impedance of the FE HZO capacitor stack is depicted in the bottom inset.

The effective parallel capacitance has been extracted at a frequency of 1 and 2 kHz respectively, since the utilized PUND waveforms are at 1 kHz and the reset pulses are at 2 kHz, Subsequently, the plot of the effective dielectric constant $\left(\varepsilon_{r}^{'}\right)$ of the HZO capacitor stack versus electric field (**Figure** **6**) allows to discern between the subthreshold region (0 < *V* < 0.3 V; in which only reversible changes of domain walls take place) and the Rayleigh region (*V* > 0.3 V; in which irreversible shifts in domain walls center of mass result in nonlinearity in the dielectric response). Based on Figure 6, we limit the amplitude of the utilized reset pulses to 0.3 V. In tandem with Rayleigh analysis, Scott et al.^[^ ^31^, ^55^ ^]^ demonstrated a notable frequency/pulse width dependence of *E*_C_ that can be attributed to the involvement of domain wall motion in the kinetics of switching. The reset pulses employed in this work are 2 kHz and polarization switching PUND pulses are 1 kHz. Pulse width variation (0.25–0.75 ms) shows *E*_C_ remains nearly constant for 0.25 and 0.50 ms duration reset pulses and change minutely for 0.75 ms (Supporting Information (i)).

## 3 Conclusion

We investigated the impact of “resetting” oxygen vacancy migration to neighboring sites in FE oxide thin films by engineering PUND‐reset pulses. We study the effect of a range of reset pulses amplitudes, which provide enough energy to allow for *V*_O_ migration between neighboring sites without inducing domain wall movement. We show the ability to speed‐up (≈1.5×) and retard (≈4× to ≈24×) the wake‐up in pristine HZO films (predominantly m‐phase), reduce rate of fatigue (in predominantly o‐phase by 4×), reduce leakage inside the films (59% difference at −2 V compared to conventional PUND), and reduce polarization loss (by 20–80%). We explain this to be related to *V*_O_ migration between neighboring sites and prove the ability to control vacancy drift to improve device performances. A comprehensive characterization is realized with in operando optical tracking of vacancies using PL and DF for films (in NPoM geometry) biased with both PUND and PUND‐reset pulses. The simultaneous delay in electrical and optical signals for PUND‐reset biased films evidently shows the proof of vacancy reset in HZO devices.

## 4 Experimental Section

4.1

4.1.1

##### Sample Preparation

Bottom electrodes (BEs) were deposited on (100)‐oriented p++ Si wafers. The Au‐based wafer was sputtered with 5 nm of TiN followed by 100 nm thermal evaporation of Au to generate the BE, while the TiN‐based wafer was sputtered with 100 nm in Ar 15 sccm/N_2_ 95 sccm environment at room temperature. ALD on both wafers produced 5 nm mixed‐phase HZO films, followed by 130 nm of sputtered Mo on a 5 inch target at 250 W gun power. Test device top contacts were patterned with UV lithography and Mo etching after a 30 s quick thermal annealing phase at 500 °C.

The raw HZO samples for spectroscopic analysis had BBI Solutions’ 80 nm‐diameter AuNPs in citrate‐capping agent drop cast on them. A density of ≈10^−3^ μm^−2^ was achieved by diluting the AuNP colloid solution. After two spin‐casting procedures, the samples were baked at 200 °C for 3 min to deposit a 210 nm layer of poly(methyl methacrylate) (PMMA). The AuNPs’ tops were then etched with O_2_ plasma to make electrical contact. Ellipsometry (J.A. Woollam EC‐400, 75 W Xe light source) revealed a final PMMA thickness of ≈60 nm.

##### Electrical Measurements

Keysight B2912A Precision source meter was used to carry out electrical measurements using custom waveforms. Measurements were conducted with a compliance current of 10 mA and a frequency of ≈1 kHz. The potential was applied to the TE, whereas the BE was grounded. Impedance measurements were performed with Solartron analytical EnergyLab XM impedance analyzer. Electrical measurements with AuNP as top contact (AuNP facet *d* ≈ 30 nm, contact area ≈700 nm^2^) were performed with a low‐noise low = current SMU. The voltage sweep resolution was set to ≈1 Hz (*V*_STEP_ = 100 mV: step directly affects sweep rates). A 100 nA measurement range and 100 nA current compliance were used. Ambient conditions were used for all measurements. Apex Probes’ SD‐qp‐CONT‐TL quartz cantilever tips were coated with 3/6 nm Cr/Au for conduction. The gap region (HZO layer between AuNP and BE) had 0.037 GPa pressure due to the cantilever spring constant, *k* = 0.01 Nm^−1^ (measured during production).^[^ ^49^ ^]^ A piezo‐controlled actuator lowered the cantilever parallel to the sample surface and stayed put during measurements.

##### Optical Characterization

The PL and DF experiment were conducted utilizing a continuous wave (CW) laser with a wavelength of 444 nm (provided by Integrated Optics) and white light illumination, respectively. The optical signal was sent by an optical fiber model QP50‐2‐UV‐VIS to an Oceans Optics QE65000 spectrometer. Spectra were integrated for 2 s for both PL and DF. PL and DF results were collected on HZO grown on Au (gold) substrate because of the enhanced prominence of the defect state associated with oxygen vacancies.

## Conflict of Interest

The authors declare no conflict of interest.

## Author Contributions

**Atif Jan:** Conceptualization (lead); Formal analysis (lead); Software (lead); Writing—original draft (lead); Writing—review & editing (lead). **Stephanie A. Fraser:** Data curation (supporting); Formal analysis (supporting). **Taehwan Moon:** Resources (supporting). **Yun Seong Lee:** Resources (supporting). **Hagyoul Bae:** Resources (supporting). **Hyun Jae Lee:** Resources (supporting). **Duk‐Hyun Choe:** Resources (supporting). **Maximilian T. Becker:** Investigation (supporting); Writing—review & editing (supporting). **Judith L. MacManus‐Driscoll:** Funding acquisition (supporting); Writing—review & editing (supporting). **Jinseong Heo:** Resources (supporting). **Giuliana Di Martino:** Conceptualization (lead); Funding acquisition (lead); Supervision (lead); Writing—original draft (supporting); Writing—review & editing (equal).

## Supporting information

Supplementary Material

## Data Availability

The data that support the findings of this study are openly available in Apollo at https://doi.org/10.17863/CAM.106565.
