# Supplementary material for: Resetting the Drift of Oxygen Vacancies in Ultrathin HZO Ferroelectric Memories by Electrical Pulse Engineering
Source: Small Sci. 2024 Jul 30;4(11):2400223. doi: 10.1002/smsc.202400223 (PMC11935283; doi:10.1002/smsc.202400223)
Supplement: Supplementary file 1 — Supplementary Material [file SMSC-4-2400223-s001.pdf]

SUPPORTING INFORMATION

Resetting the drift of Oxygen Vacancies in Ultra-Thin HZO Ferroelectric Memories by Electrical Pulse Engineering

Atif Jan[1], Stephanie A. Fraser[2], Taehwan Moon[4], Yun Seong Lee[3], Hagyoul Bae[5], Hyun Jae Lee[3], Duk-Hyun Choe[3], Maximilian T. Becker, Judith L. MacManus-Driscoll, Jinseong Heo[3], Giuliana Di Martino[1]*

[1]*Department of Materials Science and Metallurgy, University of Cambridge, 27 Charles Babbage Rd, Cambridge, CB3 0FS, United Kingdom*

[2] *Cavendish Laboratory, University of Cambridge, J.J. Thomson Ave., Cambridge, CB3 0HE, United Kingdom*

[3]*Samsung Advanced Institute of Technology, Suwon-si, 16678, Korea.*

[4]*Department of Electrical and Computer Engineering, University of Southern California, Los Angeles, 90089, USA*

[5]*Department of Electronics Engineering, Jeonbuk National University, Jeonju-si 54896, Korea.*

**gd392@cam.ac.uk*

(a) Electrical characterization of 5nm HZO films using PUND on FE Radiant Tester

We conducted a thorough ferroelectric (FE) analysis of the HZO film utilising quicker positive-up negative-down (PUND) sweeps. This characterization was performed utilising a commercially available FE tester known as Radiant. The voltage range of PUND oscillates between positive 2 volts and negative 2 volts, with a frequency of 1 kHz. The experiments were conducted utilising Molybdenum top contacts with a size of 100 µm. The Mo electrode was sputtered utilising a 250W gun power on a 5-inch target. The extracted polarisation (Figure S1(a)) was measured to be around 27 $\mu C/cm^2$. The coercive field of the films (extracted from the polarization switching current data) was observed around 0.95 V shown in the figure S1(b).

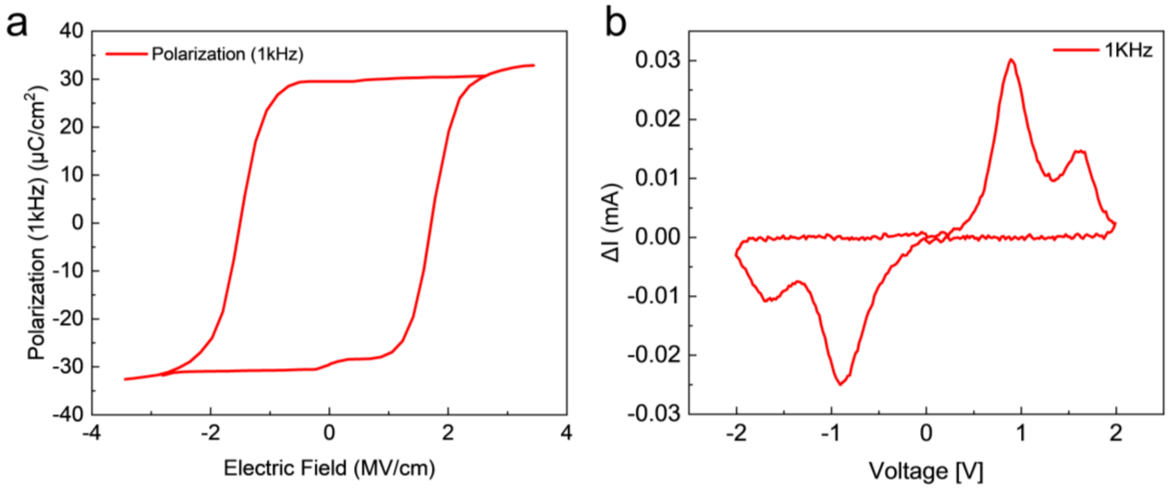

Figure S1: a) PE loop extracted on Keysight SMU on 5 nm HZO films showing remnant polarization ~29 μC/cm^2. b) Polarization current ($\Delta I = I_{P(N)}$-$I_{U(N)}$), showing the characteristics double peak for pristine films in initial cycles with coercive voltage (E_C) ~1V.

(b) Polarization vs cycles for all 9 PUND-reset

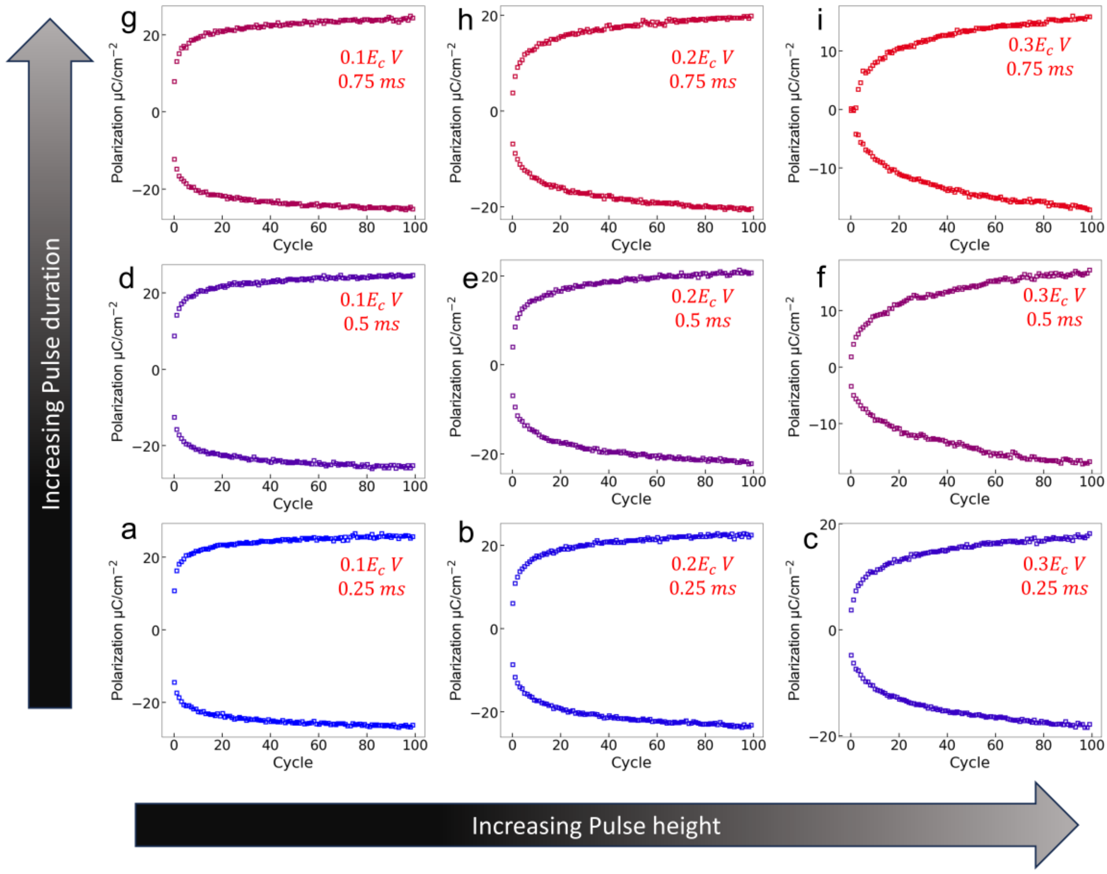

Figure S2: The polarization vs cycles for all 9 PUND-reset pulses.

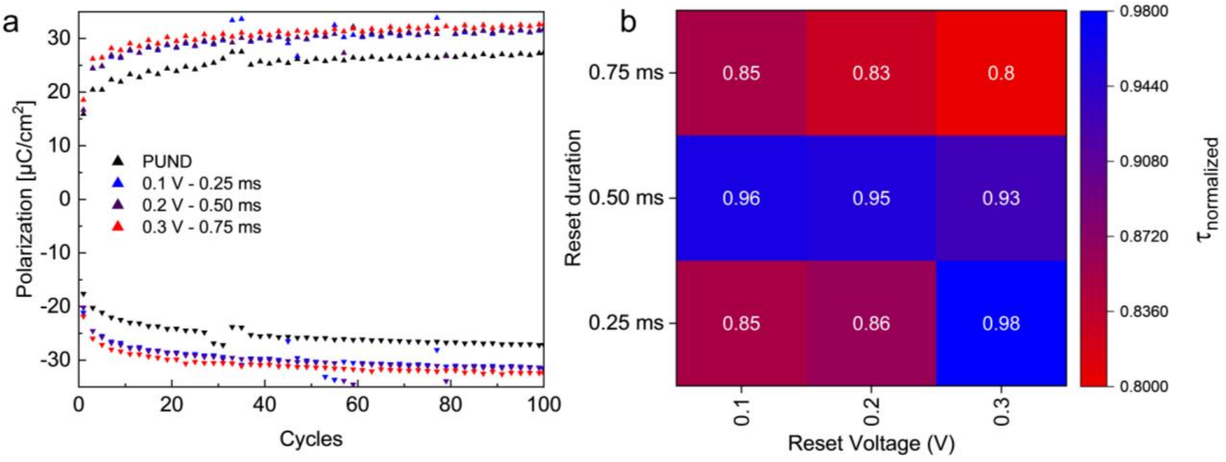

Figure S3: a) Polarization vs switching cycles for PUND (black) and three representative PUND-reset (blue: 0.1 V/ 0.25 ms, magenta: 0.2 V/0.50 ms, red : 0.3 V/ 0.75 ms) pulses. b) The characteristic time constant for nine PUND-reset normalized to that of the PUND is defined as $\tau_{normalized}$ for comparing speeding up in time to wake-up. Heatmap showing the $\tau_{normalized}$ time constants for all 9 PUND-reset.

(c) Fatigue when device cycled with PUND-reset throughout

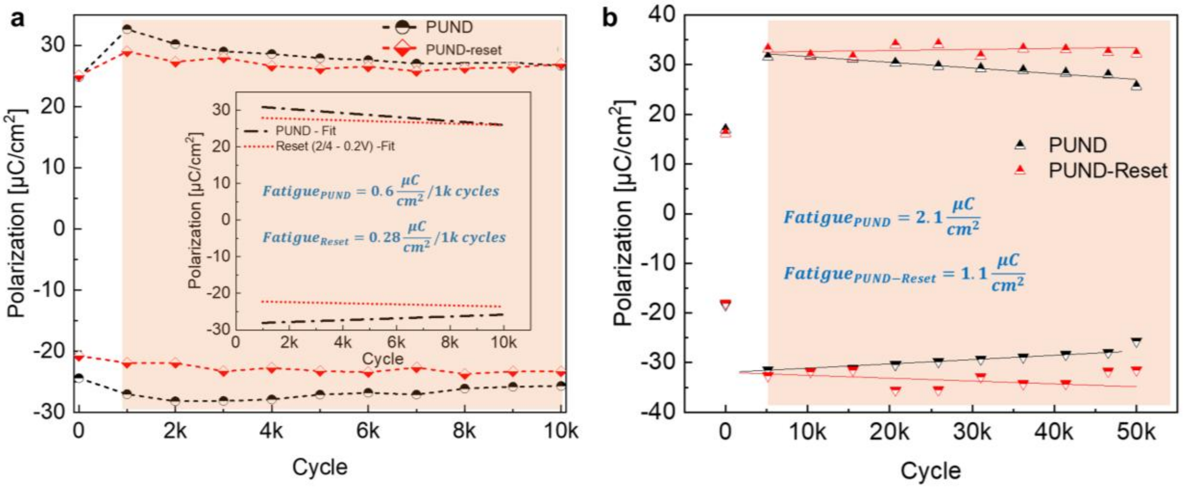

Figure S4: (a) Reduction in fatigue upon cycling the device with PUND-reset with same waveform used throughout wake-up and fatigue. PUND-reset pulses show 0.28 $\mu C/cm^2/1k\ cycles$ reduction in fatigue compared to 0.6 $\mu C/cm^2/1k\ cycles$ for PUND pulses. (b) Device with common wake-up i.e., both cycled with PUND for 2000 cycles until wake-up. Post 2000 cycles device cyled with PUND (black) and 0.1V – 0.25 ms PUND-reset (red). The

solid lines show linear fit (2000-50000 cycles, after wake-up (shaded area)) to extract slope of fatigue for each device.

FE HZO devices cycled with PUND and PUND-reset (0.2V – 0.5 ms) throughout also show lesser fatigue when reset pulses are incorporated into the conventional PUND waveforms to extract polarization.

(d) Leakage as gap in PV loop at 0 V

Assuming that interface asymmetry does not change, an increase in leakage will also result in an increase in the gap, simply because the entire loop will enlarge (Figure S5). In essence, the size of the gap is dependent on both the asymmetry of the interface and the quantity of leakage; if there is no leakage, even for asymmetric interfaces, there is no gap. It may be accurate to say that the disparity is proportional to both leakage and interface asymmetry.

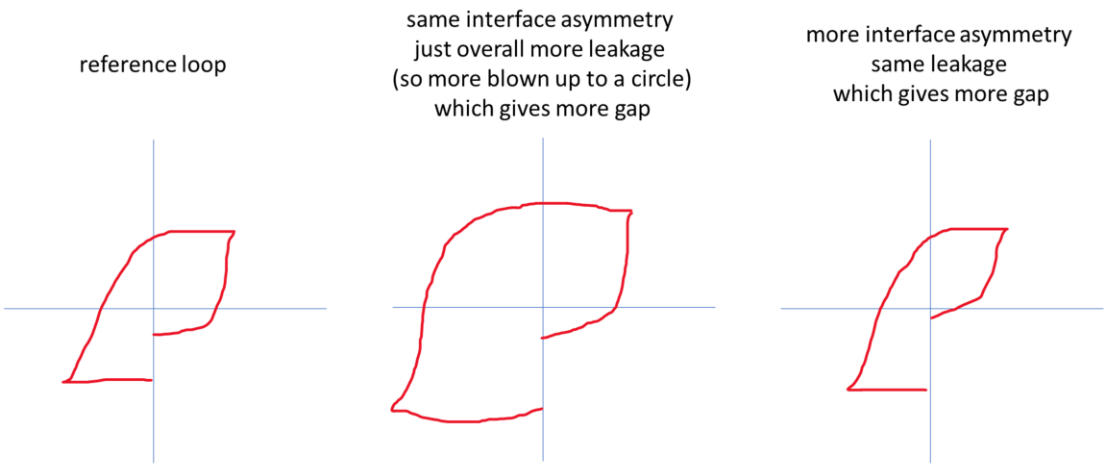

Figure S5: The reference plot (left) shows a PV loop with a gap at 0V, which could be due to leakage or device asymmetry. If device asymmetry remains same but there is overall more leakage in the film, the loop will look more blown up (middle) which will also give more gap at 0V. Similarly if the device asymmetry increases but leakage remains similar the loop will also show more gap at 0 V (right), albeit not as blown up.

(e) NPoM field cycling

Due to dynamic V_O migration in the HZO layer, PUND cannot fully adjust for leakage currents. Our device's nm-sized top contact restricts current to ~ pA, necessitating a low-noise SMU for slow current measurements (Methods). Using slower rates (~1 Hz) than

normal (100-10 kHz) can exaggerate polarisation[1]. The highly asymmetric NPoM device construction makes it hard to find a "true" device area. Thus, we depicted internal HZO fluctuations as a function of charge variation rather than absolute polarisation. The change in internal charge in the film is measured by integrating current differences (e.g., I_P - I_U and I_N - I_D) during the measurement length and normalising to the initial PUND measurement (ΔQ_0). After successive observations, HZO films show a shift in polarisation charge ($\Delta Q/\Delta Q_0$) due to internal charge[2]. Each PUND polarity (e.g., PU for +2 V, ND for −2 V) is integrated separately, therefore we centred each half around the origin for clarity. Similar films have wake-up effects, which enhance remanent polarisation almost 2x over cycling[3].

(f) Domain wall motion – Impedance spectroscopy

As depicted in Figure S6, the motion of the centre of mass of a DW in a crude energy landscape can be modelled as a sketch[4]. The Z-axis describes the spatial dimension along which the domain wall's centre of mass moves, while the E-axis describes the energy required for this movement. The blue arrows represent the reversible movement of the noncoupled or subthreshold fixation regime. The red arrows depict the movement of the region's centre of mass in its various metastable phases.

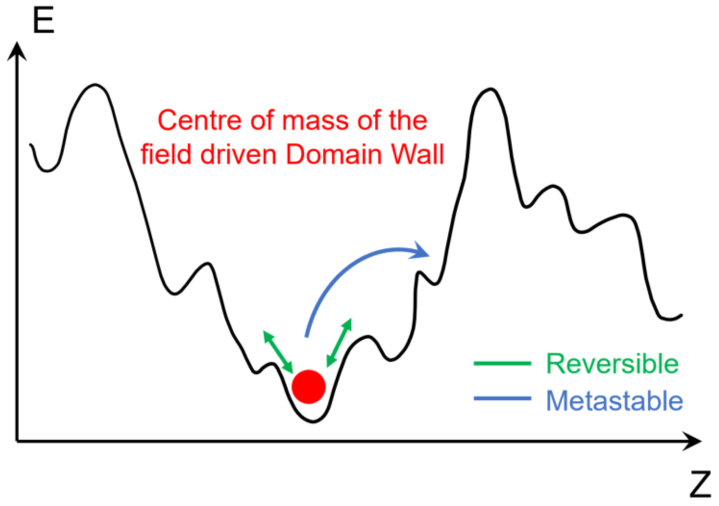

Figure S6: Sketch depicting the movement domain wall center of mass (red) in electric field landscape contrasting the movement of metastable/irreversible (blue) and reversible nature (green).

(g) Rayliegh measurement and domain wall motion

The Amplitude E_0 corresponds to the root-mean-square (rms) value of the electric field utilized in the impedance spectroscopy experiment. Since we use a sinusoidal excitation with amplitude E_1 in the impedance spectroscopy measurement. i.e., $E_1 \sin 2\pi ft$, the relationship between E_0 and the Amplitude E_1 (maximum applied field) is given by $E_1 = \sqrt{2}E_0$. Thus, the maximum applied field, “seen” by mobile interfaces in the HZO film during the impedance

spectroscopy is higher than E_0 which should be kept in mind. However, since E_0 is in a certain sense the "average" field seen by the mobile interphases, we conclude that indeed E_0 is a good measure to estimate the threshold regions for irreversible center-of-mass domain wall motion.

(h) EIS: Equivalent capacitance

Electrochemical impedance spectroscopy (EIS) has the capacity to separate complex electrochemical phenomena into faradaic and non-faradaic components. The evaluation of effective parallel resistance (R^P_{eff}) and effective parallel capacitance (C^P_{eff}) from measured impedance $Z = Z'(f) - iZ''(f)$ is as follows:

$$R^P_{eff}(f) = \frac{Z'(f)^2 + Z''(f)^2}{Z'(f)}$$

$$C^P_{eff}(f) = \frac{-Z''(f)}{2\pi f \times (Z'(f)^2 + Z''(f)^2)}$$

The R^P_{eff} values at 1 and 2kHz are displayed below

E_0	R^P_{eff} (1 kHz)	R^P_{eff} (2 kHz)
10	9760499.010981105	6575824
37.5	12605018.091936098	5997494.865789649
61.5	11323632.666872302	5305853.680390195
87.3	10352028.639250902	4964352.857586907
113	8660456.615167512	4220804.527992365
138	7098436.44394904	3879749.8206530972
164	5727679.719682904	3125038.779606147
190	4764668.003703133	2587279.010844701
216	4005583.8068656917	2101042.6735693146
242	3204996.8958052373	1676488.1684951126
267	2596030.7750795907	1355473.7475213902
293	2062606.1945397875	1080458.963341898
319	1684974.7078597238	883717.5322164586
342	1415107.96942777	740262.3640009123
371	1202033.9471873608	628306.4685206995

396	1053414.2438750407	550592.6647082225
422	959693.460211965	500106.70249689533
448	910333.8515902366	476620.724942598
474	880203.1097400009	462104.7382966553
500	818496.4462218022	435713.3761258807

(i) Coercive field vs reset pulse duration

Scott et. al[5,6], showed there is a strong dependence of coercive voltage on frequency of switching waveform. The substantial frequency dependence of coercive fields is primarily due to the involvement of domain wall motion in a viscous medium in the kinetics of switching. Viscosities exhibit a significant dependence on frequency. Ferroelectric liquid crystals exhibit frequency dependency within the range of Hz to kHz, while hard ceramics are likely to have frequency dependence within the kHz-MHz range[6]. The frequency of PUND pulses switching the polarization in our work is 1 kHz and the reset pulses is 2 kHz. We therefore, expect almost no change in E_C. Moreover, the pulse width ranging from 0.25 ms to 0.75 ms also does not impact E_C in the HZO devices. Figure S7a shows switching voltage remains nearly constant for 0.25 and 0.50 ms duration reset pulses and show a minute change for 0.75 ms.

In tandem with Rayleigh analysis, Scott et al. demonstrated a notable frequency/pulse-width dependence of E_C that can be attributed to the involvement of domain wall motion in the kinetics of switching. The E_C dependence on frequency is measured at 1kHz and 100 Hz. The difference for E_C (+) is 19% increase. While the E_C (-) is 11% increase. Assuming a linear relation between E_C and frequency[6] , the difference in E_C (Figure S7(b) for PUND at 1kHz and reset pulses at 2kHz is estimated to be around 3.8% for E_C (+) and 2.2% for E_C (-).

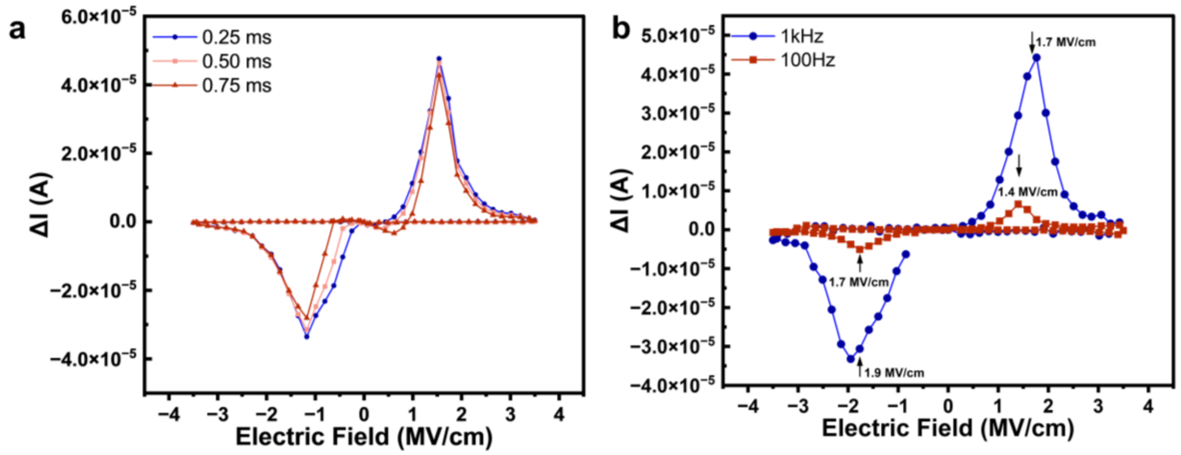

Figure S7: (a) Coercive voltage change vs result pulse duration. Coercive voltage dependence on frequency.

References:

[1] I. Fina, L. Fàbrega, E. Langenberg, X. Martí, F. Sánchez, M. Varela, J. Fontcuberta, *Journal of Applied Physics* **2011**, *109*, 074105.

[2] A. Jan, T. Rembert, S. Taper, J. Symonowicz, N. Strkalj, T. Moon, Y. S. Lee, H. Bae, H. J. Lee, D.-H. Choe, J. Heo, J. MacManus-Driscoll, B. Monserrat, G. Di Martino, *Advanced Functional Materials* **2023**, *33*, 2214970.

[3] M. Pešić, F. P. G. Fengler, L. Larcher, A. Padovani, T. Schenk, E. D. Grimley, X. Sang, J. M. LeBeau, S. Slesazeck, U. Schroeder, T. Mikolajick, *Advanced Functional Materials* **2016**, *26*, 4601.

[4] R. Marquardt, D. Petersen, O. Gronenberg, F. Zahari, R. Lamprecht, G. Popkirov, J. Carstensen, L. Kienle, H. Kohlstedt, *ACS Appl. Electron. Mater.* **2023**, *5*, 3251.

[5] J. F. Scott, L. Kammerdiner, M. Parris, S. Traynor, V. Ottenbacher, A. Shawabkeh, W. F. Oliver, *Journal of Applied Physics* **1988**, *64*, 787.

[6] "Models for the frequency dependence of coercive field and the size dependence of remanent polarization in ferroelectric thin films," DOI 10.1080/10584589608013050can be found under https://www.tandfonline.com/doi/epdf/10.1080/10584589608013050?needAccess=true, **n.d.**
